# The role of continuing medical education programs in promoting iranian nurses, competency toward non-communicable diseases, a qualitative content analysis study

**DOI:** 10.1186/s12909-022-03804-x

**Published:** 2022-10-24

**Authors:** Maryam Zarei, Sadaf Mojarrab, Leila Bazrafkan, Nasrin Shokrpour

**Affiliations:** 1 Medical Education Development Centre, Shiraz University of Medical Sciences, Shiraz, Iran; 2 School of Nursing and Midwifery, Shiraz University of Medical Sciences, Shiraz, Iran; 3 Clinical Education Research Centre, Education Development Centre, Shiraz University of Medical Sciences, Shiraz, Iran Sina Sadra Halls, Neshat Ave, 7134874689; 4 English Department, Faculty of Paramedical Sciences, Shiraz University of Medical Sciences, Shiraz, Iran; 5 Shiraz, Iran

**Keywords:** Continuing professional education, Lived experience, Nurses, Qualitative study

## Abstract

**Background:**

Continuing medical education is essential for nurses to provide quality patient care and upgrade their professional skills and competence. The need for continuing medical education (CME) has become more apparent in the face of advances in medical science, the ever-changing healthcare system, and nurses’ vital role in improving health care. It is, therefore, imperative to explore the nurses’ experience of CME courses and the extent to which such programs are effective.

**Objective:**

The present qualitative study aimed to explore and describe nurses’ experiences of the effect of CE programs in promoting their competencies toward non-communicable diseases.

**Methods:**

This qualitative content analysis study was conducted from December 2019 to April 2020 at various hospitals affiliated to Shiraz University of Medical Sciences (Shiraz, Iran) and based on the principles of conventional content analysis. The target population was nurses who actively worked in the chronic wards of these hospitals. The participants were selected using maximum variation sampling, including nine nursing managers, education and clinical supervisors, and staff nurses. Data were collected through individual, face-to-face, semi-structured interviews guided by an interview guide, and data collection continued until data saturation was achieved. Each interview took about 30–45 min. Follow up questions were used for clarification when needed. Data trustworthiness was assessed according to the criteria proposed by Guba and Lincoln.

**Results:**

Analysis of the interview data resulted in 230 primary codes, based on 8 categories, and three themes were identified. The extracted themes were gaps in the planning of the CME program, problematic context, and training to improve professional skills and competency. The associated categories were gaps in the planning of the CME program, problematic context, and training to improve professional skills and competency.

**Conclusion:**

Professional competence and performance of nurses can be improved through intrinsic motivation stimulation, planning, and implementation of training programs based on professional needs and effective assessment of the teaching/learning process.

**Supplementary Information:**

The online version contains supplementary material available at 10.1186/s12909-022-03804-x.

## Introduction

Continuing Medical Education (CME), as an essential part of the developed system of global health services, has been an appropriate approach for changing the health care behavior of healthcare providers’ educational problems within healthcare systems [1, 2]. Developing the quality and quantity in CME has long been the focus of close attention for academic centers and the capability of physicians or nurses to care for patients [3–5].

Noncommunicable diseases (NCDs), also known as chronic diseases, are of long duration and result from a combination of genetic, physiological, environmental, and behavioural factors. Decreasing the load of noncommunicable disease (NCD) is a universal development imperative. The main types of NCD are cardiovascular diseases (such as heart attacks and stroke), cancers, chronic respiratory diseases (such as chronic obstructive pulmonary disease and asthma), and diabetes [6–8].

Investments in the prevention and control of NCDs bargain a high return for countries at all income levels; in the long term, NCD prevention offers a higher return on investment than NCD control, though both are vital to an effective reaction strategy [9].

A large group of health care professionals make a vital contribution to tackling NCDs and are the key providers of NCD prevention, treatment, and management. As the point of first contact, nurses are well positioned to detect, treat, and refer the patients with NCDs and provide information, education, and counselling to the public on the prevention of NCDs [10–12]. With their holistic approach to care, nurses are well prepared to provide behavioural and lifestyle interventions that consider the social determinants of health and build on the strengths and resources of the individual and his/her community. Patients are encouraged to increase their health literacy and the ability to understand and act on health information, so that they get empowered to become active participants in their care [13]. Nurses can advocate for the policies integrating NCD prevention into health planning and as role models for healthy lifestyles [8]. Unhealthy lifestyle choices are a risk factor for NCDs; positive behaviour change is possible through awareness and education. Research has demonstrated that nurses effectively support individuals to make behaviour changes, administer disease management programs, and enable self-care and self-management. Practicing environmental issues that prevent the nurses from fully reaching their potential in addressing the NCD crisis needs to be addressed [13–15].

For instance, cardiovascular and chronic lung diseases, stroke, and type 2 diabetes are among the top 6 leading causes of death in the Eastern Mediterranean region and Iran. The prevalence of these diseases in Iran is exacerbated by urbanization, changes in lifestyle due to industrialization, changes in age composition, and population aging [14–16].

Providing quality health care and meeting the patients’ psychological, social, and spiritual needs positively affect their acceptance of the illness, self-esteem, and life expectancy [17].

Universities of medical sciences in Iran are responsible for the population’s health for a defined area, such as educating the care providers to prevent NCDs. The Deputy Minister of Health and Medical Education developed the prevention and control package for education and decreased the related risk factors. This package, entitled Iran’s Package of Essential NCD Interventions (known as IraPEN), is being implemented throughout the country according to the trend of change in the health sector and has received the top priority in the health system of our country. The healthcare providers, including nurses at healthcare centers, provide this package [18, 19]. Nurses, as essential members of medical teams, should also receive continuing medical education (CME) to keep pace with advances in therapeutic processes to contribute to patient care efficiently [18, 20].

CME programs in Iran usually involve traditional and sometimes ineffective approaches [20, 21]. In Iran, studies have identified various problems, such as the poor quality of the CME in nursing. This shortcoming is duo to the lack of attention to the nurses’ educational needs and poor administration of the educational programs, lack of coordination between the programs and the field of activity, insufficient attention to professional demands and the educational content, inappropriate teaching styles, and ineffective programs [18, 22–24].

However, a previous review study on the concept of CME courses has indicated that in-service training of health care personnel enhances their knowledge, skills, motivation, and attitude, positively affecting their self-esteem, performance, and job quality [20].

In addition, the recent Covid-19 pandemic and the imposed sanctions have introduced further challenges to the healthcare system in Iran. Decreasing the financial resource and prioritizing Covid-19, in turn, has diverted educational focus from noncommunicable diseases. However, neglecting NCDs in this era could potentially lead to a calamity post-Covid [25, 26].

In Iran, due to the role of noncommunicable diseases in increasing mortality in society, and based on the functions and policies of the Ministry of Health for capacity-building and the instructions for compulsory education, it has been decided to include the topics related to noncommunicable diseases in all educational programs, including undergraduate and postgraduate programs as well as CME.

Despite the long history of holding and applying continuing education for the nurses, we have no experience in continuing education in NCDs among nurses. Also, no positive effects are observed in improving the nurses’ professional function and promoting the nursing care quality in NCDs. Conducting a qualitative study can help better understand the social context and individual’s perception of the obstacles, facilitators, and nurses’ participation in the CME program. This research aimed to describe the nurses’ experience in the CME program and understand why and how they become proficient, and why they are not competent in noncommunicable diseases, which is the most concern of stakeholders in medical education to underline the role of continuing medical education.

## Methods

### Study design

This qualitative content analysis study was conducted from December 2019 to April 2020 at various hospitals affiliated to Shiraz University of Medical Sciences (Shiraz, Iran) based on the principles of conventional content analysis by deductive and interpretive approaches. This method contributes to deep exploration of experience and understanding of the data, leading to conclusions about the meaning of these experiences [27].

### Setting and sample

The target population was nurses actively working in internal wards of various hospitals affiliated with Shiraz University of Medical Sciences (Shiraz, Iran). These hospitals have a history of providing 70 years of health care service.

The participants were selected using purposive sampling with maximum variation in terms of sex, education, rank, and two years of work experience; the nurses had to undergo continuing education with educational supervisors, head nurses, staff nurses, and all continuing education program-related workforces. Firstly, in the present study, the researcher referred to each university hospital affiliated with Shiraz University of Medical Sciences to explain and clarify the project to the educational supervisors. They were responsible for providing continuing education in hospitals. They were asked to introduce the nurses who met the inclusion criteria.

The sampling continued until data saturation, when no new information could be extracted [28, 29]. The inclusion criteria were at least a bachelor’s degree in nursing, ≥ 2 years of work experience, mental and physical ability to participate in an interview, and willingness to participate in the study. The exclusion criterion was the nurses who had not presented the CME program related to non-communicable diseases. Accordingly, a total of 8 individuals, including a chief nurse (n = 1), education supervisor (n = 2), clinical supervisor (n = 1), head nurse (n = 1), CME agent [1] and staff nurse (n = 3) were recruited. The demographic characteristics of the participants are presented in Table 1.

**Table 1:** Demographic characteristics of the participants

Participant	Sex	Age (years)	Marital status	Education level	Function	Work experience (years)
P1	Female	44	Married	BSc	Chief nurse	15
P2	Female	48	Single	MSc	Education supervisor	16
P3	Female	39	Married	PhD	Education supervisor	12
P4	Male	31	Married	MSc	Clinical supervisor	7
P5	Female	42	Married	MSc	Head nurse	15
P6	Male	35	Married	BSc	Staff nurse	6
P7	Female	38	Single	BSc	Staff nurse	12
P8	Female	45	Married	BSc	Staff nurse	20
P9	Female	48	Married	MSc	CME agent	20

### Data collection

A total of 9 individual, face-to-face, semi-structured interviews were conducted using an interview guide (Table 2). The interviews were held at a pre-arranged time in a private and quiet location at the participants’ workplace. The interviews started with open questions and were followed by other related questions. Each interview took about 30–45 min. Follow-up questions were used for clarification when needed. MZ conducted the interviews under the supervision of LB. Audio recording of the interviews was done with the prior permission of the participants. Each interview started with a set of open questions, e.g., “*What is your impression of the continuing education course that you followed in* NCDs ?“, leading to detailed questions to extract extra information and clarification. After holding the interviews, in case of additional questions, the researcher communicated with the participants by referring them by phone or Email concurrent to data collection.

**Table 2:** Semi-structured interview guide

Interview guide	
1	Which CME courses have you attended so far? Which topics were covered? How did you experience the training courses?
2	Please give an example of a CME course that has improved your knowledge, skills, and attitude (e.g., cardiovascular disease, cerebral palsy, trauma, addiction, etc.)
3	Which CME course was most effective? Why?
4	Considering the high prevalence of non-communicable diseases, what are the main challenges in providing nursing care?
5	Based on your experience, what needs to be done to make a CME course more effective?

### Data analysis

The conventional content analysis method was used to identify the themes from the interview data. Content analysis of the data was performed using the five steps proposed by Graneheim and Lundman. After each interview, the audio recording was transcribed verbatim and read several times to immerse in the data and understand its content thoroughly. Then, the words, sentences, or paragraphs which contained important information were identified and classified as semantic units from which primary codes were extracted. These codes were then compared, merged, and grouped in terms of similarities and differences to identify the subcategories. Categories were similarly extracted from the subcategories, from which the themes were subsequently identified [30, 31]. Data organization and analysis were performed using MAXQDA 2007 software.

### Rigor

Various criteria have been proposed to determine trustworthiness in qualitative research. However, Guba and Lincoln propose the most comprehensive criteria: credibility, dependability, conformability, and transferability [32, 33]. The credibility criterion was fulfilled through prolonged engagement of the research team with the study, verification of transcripts to confirm statements made by participants, allocation of sufficient time to build rapport with the participants to obtain accurate information, and confirmation of the information through member checking. Dependability criterion was fulfilled through peer debriefing and review of the data analysis process by the research supervisor. Conformability criterion was fulfilled through verbatim transcription of audio recordings and reconfirming the transcripts, semantic units, and primary codes with the participants; also, the transferability criterion was fulfilled through purposive sampling of the participants with maximum variation [33].

## Results

Analysis of the interview data resulted in 230 primary codes, based on 8 categories, and three themes were identified (Tables 3 and 4).

**Table 3:** ; Examples of extracting codes, categories, and themes from raw data

Meaning unit	Code	Category	Theme
“Patients expect me to receive care according to professional standards of nursing practice and require care to be conducted correctly and appropriately. I did not understand the speaker’s meaning in a CME course, and I did not know the reason for holding these programs”	Lack of attention to Patients’ needs Lack of attention to nurses’ needs prior to designing the program; Lack of interaction with participants	The gap in knowledge contents	Gaps in the planning of the CME program

**Table 4:** Extracted themes, categories, and sub-categories from the interview data

Themes	Categories	Subcategories
Gaps in the planning of the CME program	The gap in knowledge contents	Need assessment, patients need, learner needs, society needs, Providing content regardless of important factors such as non-communicable diseases Providing content regardless of important factors such as mortality of diseases Lack of attention to Patients’ needs Participants did not differentiated non-communicable diseases from another subjects. Hospitalize patients is not the of a real sample of patients in society
	presentation methods	Teaching methods, problem based learning, small group teaching, interactive learning. Passive learner, False audience, Lack of attention to the audience poor quality peresentation instructors teaching outside their expertise, presenting outdated topics traditional teaching methods
	evaluation of the CME program	Lack of giving feedback after the program Need for evaluating the nurses after the program Need for evaluating the nurses after the on work place
problematic context	work overload	Nurses work load Increase work load due to diseases load Neglects non-communicable diseases due to communicable diseases (covid) Lack of communication for planning a program
	the educational environment,	Improper educational environment, Administrators’ lack of criteria for selecting instructors obstacles during training the shortcoming resource Getting credits as a motivation for participating participate for credits
	resource shortcoming	social conditions, imposed sanctions Decreasing the financial resource Prioritizing of finance high prevalence of non-communicable diseases high prevalence of non-ommunicable diseases poor facilities
Training to improve professional skills and competency	Motivation	Ignore the nurse’s knowledge and competency by instructors Motivation for participation Motivation for best practice
	self-efficacy	vicarious experiences of nurses, Self-directed, self motivated , emotional arousal. higher performance accomplishments , self-regulation of refractory behavior, mastery experiences, that nurses who faced a challenge, feel a sense of accomplishment . Social modelling. Imaginal experiences. High self-efficacy can help you lead a healthy lifestyle. Resilience to stress, Improved employee performance, Educational accapacity

### Theme 1: gap in the planning of the CME program

Based on the participants’ experiences, the categories associated with this subtheme, a gap in knowledge contents, presentation methods, and evaluation of the CME program, must be correct to improve the clinical knowledge and professional attitude of nurses in NCDs subjects. Training to improve clinical knowledge was strongly recommended as a prerequisite for effective nursing care which was directly associated with faster recovery of patients. However, the participants indicated that improving clinical knowledge and the competence of nurses necessitates understanding the audience’s needs regarding medical problems. This need assessment should be the critical item in such training. Furthermore, they stated that the main requirements for effective nursing care were gaining up-to-date knowledge, applying evidence-based nursing practice according to professional standards, and anticipating unforeseen situations that require immediate resolution. For understanding the needs of nurses before implementing the program, it is necessary to interact with them to understand their real needs. Some participants’ experience implies a lack of interaction and understanding; others have considered holding programs in line with their educational needs. However, the participant must first feel the need to participate in the program and know the goals of the program. One of the participants stated:

“*Patients expect to receive care according to professional standards of nursing practice and require care to be conducted correctly and appropriately. I did not understand the speaker’s meaning, and I do not know the reason for holding these programs?“* [P1]

One of the nurses expressed the experience of confrontation with the problems of the patient after the CME program as support as follows:

“After participating in a sound continuing educational program on nursing, a diabetic patient in emergency conditions, I recognized that a patient’s reaction to minor medical issues is inherent to nursing care, which requires knowledge and skill. For instance, I immediately suspect low blood glucose levels as soon as a diabetic patient becomes aggressive.“ [P2]

The participants expressed that attitude outweighs knowledge, and skill-based attitude contributes to change. Consequently, the needs assessment is a critical stage of curriculum planning. The curriculum developer should investigate the knowledge and attitudinal needs and seek to change people’s attitudes because it is the attitude that determines the behaviour. A participant stated:

*“Continuous education throughout our professional life empowers us to practice nursing care based on modern scientific principles such as noncommunicable diseases. Without such a learning attitude, we cannot provide optimum care and will risk physically harming our patients because noncommunicable diseases develop over time and need to be trained to improve their lifestyles. If I believe in something, I will act. For example, there was an elderly lady who needed care at home after being discharged from the hospital; I took care of her for two hours a day for two weeks.“* [P9].

Categories associated with this subtheme were the incorporation of training fundamentals for effectively transferring knowledge and recognizing common challenges and barriers to effective training. A practical training course requires a well-thought-out plan to create a high-quality program, proper implementation, and practical evaluation of the teaching/learning process. The first step in formulating a training program is to identify the specific needs of the target population. A participant stated:

“*There is a huge difference between developing a training program for adults and children. The attention of adults is attracted when the program addresses their questions and covers their immediate needs. Current training evaluation forms are traditional surveys and do not assess the need fulfillment of participants’ knowledge and skills in relation to new needs such as the NCDs subjects .*“ [P7]

The participants emphasized the importance of proper implementation of a training program. Learners are more motivated to engage in and complete the course if the instructor presents up-to-date information, uses interactive learning techniques, and initiates group discussions. A participant stated:

“*I only have good memories of those training courses that were lively, interactive, and included Q&A sessions or discussion groups. I always remember these instructors and what they taught me.*“ [P1]

The participants indicated the importance of evaluating a training course to assess its effectiveness. Feedback from learners is essential to determine the effectiveness of the courses, the instructor’s competence, and the extent of knowledge or skill acquisition by the participants. Two participants stated:

“*Post-training evaluation is an essential part of any training course. The organizers must evaluate the program and know how much we have learned. As a supervisor, I always praise the nurses who help and teach chronic patients and follow up on their problems.*“ [P6]

”*What helps me to remember what I have learned from a training program is Q&A sessions, group discussions, and, most importantly, the feedback evaluation. In one of the refresher CME programs, the teacher asked a question about asthma, which I answered incompletely; the teacher kindly completed my answer; I always remember the details of this issue.*“ [P5]

Some nurses’ experiences indicated that CME had no effects on their future work, and they did not trust this program would lead to the competence and capabilities of nurses or any progress in caring for the patients. Moreover, CME does not meet the need of nurses and patients. Thus nurses remain separate from their professional needs, and patients have not been given the care and education related to their disease. One of the participants stated:

*“We know what the problematic issues in nursing practice are. They are not only* NCDs subjects, but also all of the knowledge and skills important in nurses’ duty. *New graduates do not have accurate information about drugs. I once saw that one of these nurses, wanted to injecte the drug into the patient’s vein(IV) instead of the muscle (IM).“* [P6].

### Theme 2: problematic context

The context dimensions affecting the promotion of the nurses’ competency about noncommunicable diseases were classified into three subcategories: the timing in the performance of the nurses in the workplace, work overload, educational environment, and insufficient resources.

The timing was a typical obstacle to practical training, especially when training sessions coincided with participants’ professional/personal duties. The effect was amplified by instructors teaching outside their expertise, presenting outdated topics through traditional teaching methods, and having poor facilities. These, in turn, led to decreased motivation to actively participate and learn. In the worst-case scenario, a course was attended to obtain the required certificate or fulfill compulsory attendance. A participant stated:

“*Nothing is worse than attending an evening training course after a long day at workfor long lectures in a setting lacking ventilation and proper facilities.*“ [P8]

Participants in this study further experienced that shortage of personnel, deficiency of equipment, and unequal nurse-to-patient ratio always have adverse special effects on the patients’ care. For example, one of the participants said:

*“As the remaining facilities in this situation (sanction and Covid-19) increase the number of patients, nurses have difficulty taking care of patients and doing duties because the cost of managing Covid patients is hefty. Especially, shortage of expert nurses and workload all have a negative effect on the nurses’ care.“* [P9]

Another participant said: “*The pressure of work and overloading of responsibilities make the nurses so busy that they seldom have enough time to participate in the CME program, so I am not up to date and cannot provide excellent care based on the current evidence*.“ [P4]

Participants claimed that permanent employment in nursing was an important positive factor in contributing to the CME program to increase their knowledge, skill, and experience in nursing care and being more accountable and responsible toward patients. According to the participants, nursing care should be based on the well-established nursing process, a scientific method to provide quality care that requires an expert nurse. A supervisor participant stated:

“*The nursing process helps us to remain focused and plan timely care despite busy schedules. This care is critical in the case of chronic patients with multiple issues. How is it that novice nurses are less motivated in any education? because they are not permanently employed”* [P8].

The participants mentioned many experiences which highlight resource limitations for patients and the nurses. One staff nurse said:

“ *We have good knowledge and attitude, but practical nursing care needs equipment and resources; attitude alone does not work. According to sanctions and Covid-19 pandemic, all the resources, personnel, staff, or other facilities are moving to the Covid ward. There have been conditions in which we have had five ICU cases and seven head injury patients. How can nurses offer adequate care when needed in these situations? .“* [P9].

The participants expressed the lack of property for educational programs due to inadequate funds as one of the main obstructions to participation in continuing education CME programs. Providing the program budget is one of the most critical problems for program originators. In addition, nurses objected to paying registration fees independently. In this regard, one of the expert nurses stated:

“…*Expenditures of designing and implementing an appropriate educational program are very high. These expenses include the instructors’ tuition fees, catering, and providing equipment and educational facilities. Moreover, nurses do not intend to pay for registration fees for obligatory continuing education program, while the hospital should provide all the expenses*…” [P7].

Based on the participants’ experiences, nurses do not live in an imaginative region; their practice and patients care is affected by some external contextual factor such as cultural, social, and political pressures, whereas the lecturer focuses on the diseases in their experience; this is a formal type of education.

A young nurse said:

*“Once a farmer died due to hypertension and CVA (cerebrovascular accident). When I asked his daughter how he used the drugs, she said whenever we could find them; my mother is also sick, and we did not have enough money to pay for two patients at home. If you take care of patients in society, they face family, economic, cultural, social, and many other issues, especially in* NCDs; y*ou cannot go and teach them separately.“* [P3].

Another problem and reason to participate in CME constituted the busy life of participants. Nurses’ reasons for actively participating in CME ranged from mere motivation and performance improvement to simply getting the credits to fulfilling legal frameworks. Two associated concepts delineate this issue: motivating factors and legal frameworks. For instance, A participant stated:

*“My participation in CME is more a legal requirement than a feeling of need; I mean that if I want to practice in nursing, I have to go through the course. I do not believe that taking the course is of benefit to me.“* [P4].

### Theme 3: training to improve professional skills and competency

This theme relates to a gradual transformation from a novice to a competent nurse through effective continuing education programs with two subthemes, motivation and self-efficacy in acquired competency. Awareness and enthusiasm contribute to the promotion of the nurses to help the patients and act in a self-directed manner when faced with problems, such as noncommunicable diseases. The motivation and encouragement of nurses to educate themselves and participate in the CME program is described as a willingness to make a difference in the future health of society. Nurses find it essential to heal, comfort, and direct patients and their families, increasing their well-being. Thus, the self-directed approach drives the desire of nurses to become professional and competent.

One of the participants noted:

*“To train me better, the authorities should know what I am interested to motivate me, and which area is important for me. I was greatly supported in self-confidence during self-learning and participation in the CME program.“* [P9]

Another participant said:

*“Working in the internal department allowed me to learn more about noncommunicable diseases. As a human, I better learn what I practically do and experience.“*

Based on the experiences of the participants in the study, in addition to internal motivation, participating actively in continuing education programs; learning subjects such as communication skills with patients, principles of patient education and principles of counselling; and having sufficient information about noncommunicable diseases are necessary to acquire competence.

Based on the participants’ experience, optimal care can be achieved through effective nurse-patient communication. Therefore, nursing care is based on mutual trust and verbal and nonverbal communication techniques. For covering various facets of patients’ needs, holistic and person-centered care includes aspects such as physical and mental health needs, spiritual care needs, facilitation of the needs of dying patients, follow-up care, and guidance of patients toward recovery. A participant stated:

*“Patients suffering from chronic diseases experience physical, mental, spiritual, and financial issues on top of having to deal with discouraging news about their illness and family frustration. They are constantly anxious about the course of their illness and face the fear of death. Mere fulfilling their physical needs without addressing mental care needs may negatively affect their recovery process and lead to severe depression. These patients should receive comprehensive care. I always try to be a good listener to these patients”* [P5]

Participants who had already benefited from continuing education programs expressed the importance of gaining nursing skills and learning from the success stories of fellow nurses.

*“One of my patients with a heart disease requested medication due to stomach pain and nausea after having had a cheese sandwich for dinner. Based on my experience, I suspected a potential heart issue; I immediately informed the physician and initiated ECG and cardiac enzyme tests. A cardiologist was called upon, but the patient suffered a heart attack. Luckily, my suspension saved his life because of timely diagnosis and intervention.“* [P7]

Training to improve the professional attitude of nurses was related to the observance of the Patients’ Rights Charter, a humane attitude toward patients, the importance of time management in providing quality nursing care, and the prevention of harm to patients. The underlying philosophy of such training should be based on the spiritual narrative indicating that “whoever kills one life kills mankind entirely, and whoever saves one life saves the entire mankind.“ A participant stated:

*“It is important to train nurses, particularly novice nurses. I do not differentiate non-communicable diseases from another subjects to respect the patients’ dignity and implement the Patients’ Rights Charter. Essential in nursing practice is to treat patients respectfully, respect their rights and preferences, request permission before conducting medical procedures, and respect their privacy and confidentiality. For example, making fun of an obese patient is unethical.*“ [P4]

According to the participants, advances in medical sciences and information technology necessitate continuing education to be competent in noncommunicable diseases and other subjects. Nurses should keep up-to-date with such developments as well as attend continuing education programs. A participant stated:

*“It is natural to lose skills or forget certain information over time, either due to the lack of practice or the aging process. Continuing education plays a pivotal role in overcoming these. Today, we have advanced technology against obesity and how to diagnose diseases quickly, for example heart disease and cancer which we should be aware of and include them in educating patients and nursing care”* [P3]

The participants believed that an experienced nurse should immediately focus on the patient’s health problems and attempt to solve them without delay. Such nurses sense critical conditions, are mindful of inpatient care, are vigilant, and anticipate the occurrence of any complication associated with a disease. They are ready to take immediate action to resolve a problem and prevent potential patient risks. One gains these capabilities through a positive attitude towards learning the latest developments in nursing science. A participant stated:

*“Anticipating a patient’s health problems is an integral part of the nursing profession, which requires extensive knowledge. For example, I know when a diabetic patient becomes aggressive, low blood sugar levels should be suspected.“* [P2].

In this sub-theme, the patterns of capability development described by words such as self-directed learning and self-efficacy are considered. At this point, the nurse tries to follow self-directed learning, and the amount of time allocated to expertise acquirement seems to be one of the most critical factors. In this regard, one stated:

***‘’…****I participated in several program (CME)().My achievement in this job (nursing )) is, first of all, due to self-effort and self-learning. For example, to take full accountability, I think about everything interrelated to the take care of patients, and when I feel the need to accomplish my task, I do my best and use every possible program to empower myself, including CME****…”*** [P2].

## Discussion

Nurses play a critical role in the healthcare system. Their clinical competence is known to be directly related to continuing professional development. Hence, we aimed to assess the factors that influence the effectiveness of continuing education in improving the knowledge, skills, and attitude of nurses caring for patients with noncommunicable diseases. Analysis of the interview data resulted in the identification of three themes, gaps in the planning of the CME program, problematic context, and training to improve professional skills and competency (Table 2).

### Gaps in the planning of the CME program

According to the participants in this study, the first dimension was the gap existing in the planning of the CME program. This gap can be seen in all components of CME programs and means that the presented content, teaching method, and evaluation of the learning of the audience and program in CME are inappropriate and need to be revised. Deficiencies in the CME component and the need to revise educational programs have also been confirmed by other studies [22–24]However, based on the participants’ experience in this study, there are some gaps in the planning of the CME program related to noncommunicable diseases, such as presenting the program and evaluating the learning of audiences.

Our results are similar to those of Zargham et al. (2013), who, in a qualitative study, found the educational challenges that emerged from inaccurate curriculum training development and the lack of consideration of the learners’ attitudes [34].

The critical point here is that although the CME program from an administrative and organizational perspective is represented by need assessment, nurses often assess their program by their real needs. In order to understand the needs of nurses before implementing the program, we need to interact with them to understand their real needs. In this line, Griscti and acoJno state that CME programs should be developed under the needs of the target population, incorporate the fundamentals of adequate training, and always include effective evaluation of learners. They conclude that such courses are only effective if the needs and expectations of learners are fulfilled [35]. Similarly, Chong et al. stated that training programs should at least meet the nurses’ minimum educational and professional needs [36].

Laatikainen et al. emphasized that capacity-building intervention improved the capacity of professionals to detect and manage noncommunicable disease risk factors [37].

Improving knowledge is one of the critical success factors in nursing care. The participants believed that providing evidence-based nursing care indicated improved nursing knowledge. However, some studies have pointed out gaps in the continuous education programs, such as defects in the content, teaching method, or evaluation of the desirable and current situation [37, 38]. Instructors of training courses are the main contributors to the effectiveness of training. In addition to the importance of good content, teaching techniques that increase participation contribute to training effectiveness. In a study conducted in Australia on educational needs, Booth and Lawrence highlighted the importance of selecting a training method based on comprehensiveness and active learning principles [39]. Davis et al. have reported the superiority of interactive courses that increase participant engagement (e.g., workshops, group discussions, and even participating in clinical rounds) over lecture-based refresher courses [40]. In two other studies, White et al. reported positive results from workshop-based retraining courses based on problem-based learning methods [41]. In line with our results, Jalali showed that 22% of their study participants stated the importance of presenting up-to-date information during CME courses [42]. Islamian et al. reported that almost all their study participants stressed the importance of using experienced instructors for a successful instructor-led learning experience. They also stated an inherent conflict between inappropriate teaching methods and practical learning [20].

Program evaluation and training effectiveness are judged based on the learner’s knowledge and the extent to which skills are developed practically to benefit the organization concerned. Therefore, it is essential to observe the fundamentals of practical training, which include planning and developing an effective program, proper implementation, and effective evaluation of the teaching and learning process. There is also a need to recognize challenges and barriers to effective training promptly and overcome these issues as the training progresses, in line with our findings [43–45]. Nevertheless, Islamian et al. stated that an assessment of the effectiveness of training programs was not conducted correctly in Iran [20].

Evaluating participants in continuing nursing education programs is also essential and should be appropriately planned when designing training programs. The study by Aminoroaia et al. (2014) explained that participants prefer periodic assessments, strict supervision of assessments, assessment of the effectiveness of training, and conducting knowledge tests both before and after the completion of the training program [45].

### Problematic context

According to the nurses’ viewpoints, context dimensions affect the promotion of competency toward noncommunicable diseases from the nurses who address challenges and overcome barriers associated with effective CME. The timing in the performance of the nurses in the workplace, educational environment, and shortcoming of resources are the subthemes that emerged from the data indicating the importance of context.

Most participants indicated work overload as one of the most critical obstacles to effective participation in the CME education program. These results are consistent with those of other studies, such as the research conducted by Shahhosseini and Hamzehgardeshi (2015), in which two main levels of updating information and professional skills were extracted as the most common facilitators and workload and lack of support as the most common barrier to nurses’ participation in continuing education program [46].

Regarding the obstacles, as in the case of Zamanifar, Asgari (2022) and Masoumi et al. (2019 ), lack of time was the main barrier restraining involvement in CME programs, which was in line with our study [47, 48].

The present study showed that the shortage of educational assistive equipment and treatment facilities in hospitals and the shortage of educational budget were among the other challenges for CME programs. Chong (2011) pointed to the costs of continuing education programs as obstacles to the nurses’ attendance in continuing education programs [36]. Farmani et al. (2009) who reported the nurses’ point of view related to continuing nursing education revealed average fulfillment with the place of educational classes. Similarly, the nurses also believed that facilities for attendance of learners in different shifts in educational classes are essential for the promotion and efficacy of continuing education programs [49].

In a previous study, Zang and Petrini (2008) reported that time constraints due to job or family responsibilities were the main obstacles for nurses to participate in CME courses [50]. Another study also reported that job responsibilities and subsequent lack of time were the main obstacles to participation, resulting in the importance of CME being downplayed [46–48].

In other studies, the managers’ critical role in learning and facilitating the nurses’ promotion toward professional competency were indicated as one of the main factors of care quality. Most content analysis studies have emphasized this issue and reported the disinterest of authorities as the reason for the low efficiency of continuing clinical education [51–53].

### Training to improve professional skills and competency

The present study findings indicate that nurses require a combination of theoretical education and practical insight related to noncommunicable diseases based on their needs by an actual method of instruction and assessments. Based on their experiences, continuing education on the patients’ needs, such as noncommunicable diseases, is essential and should be implemented regardless of the current restrictions resulting from sanctions and subsequent lack of resources. Otherwise, the costs to the national healthcare system will be overwhelming and negatively impact patients due to untrained staff. Those with non-communicable diseases are mainly middle-aged and elderly patients, whose diseases reduce the quality of life of the involved family members. Nurses must be equipped with the necessary knowledge, skills, and capacities to deal with the problems and challenges associated with noncommunicable diseases. In this regard, continuing education will improve the current inappropriate approaches and deliver the best results. [20]. Change in attitude is of primary importance, followed by the need to improve skills and knowledge in the later stages because attitude creates the context for acquiring knowledge and skills [54]. McDiarmid (2011) stated that participants in their CME courses were highly motivated to increase their professional knowledge to improve the patient care [55]. Keshmiri et al. reported improved quality of nursing care by those who participated in their especially designed CME courses based on the competency model [56].

The participants described improving professional competence and skills as essential to optimal nursing care. Clinical skills encompass all aspects of care, such as holistic care (physical, psychological, and the needs of dying patients), physical environment management, and guidance to hospitalized patients from admission to discharge. Some et al., in their descriptive study, state that nurses can follow protocols for managing noncommunicable diseases of patients based on perfect and standardized protocols and guidelines; shifting nurses to help to manage noncommunicable diseases fills the significant healthcare gap in developing countries [57].

Dewar et al. describe a nurse as a person that pays attention to all care needs of patients, even minor issues and routine care [58]. Boswell et al. (2013) also emphasized the patients’ spiritual care needs of [59]. In a previous study, Watson suggests that proper nurse-patient communication to gain the patients’ trust is integral to nursing knowledge and skills. In other words, nurses should present themselves in a manner that gives patients confidence in their knowledge and skills [32].

Qanbari Qalehsari et al. (2017) identified in their research that educators could not only help to maintain the zeal of nurses who are willing to learn, but also assisst those who are less motivated if they use more learning-centered approaches when designing lifelong learning programs [60]. Moreover, they concluded that typical challenges associated with teaching and learning processes were inappropriate knowledge transfer methods, lack of motivated instructors, and unfamiliarity with the latest teaching methods [33]. A study carried out by Farmani and Zaghimi indicated a moderate match between the content of CME courses and participants’ professional needs [49].

The use of distance learning is one solution that can increase the nurses’ participation in the CME program. Some studies point out that nurses perceive the benefits of e-learning on their personal and professional development; higher self-efficacy and performance scores were generally found among nurses using the e-learning intervention, and nurses improved their confidence in reducing stress in the nurse-patient relationship [61–63].

Interestingly, the training results in the CME program are slightly contradictory. The nurses appreciated the program; on the other hand, education seemed to remain a simple process with some problems. Although the program enhanced the nurse engagement and participation, the learning did not occur on a practical and deeper level due to the absence of self-efficacy, and lack of clear learning objectives related to the actual needs of nurses could explain the paradox and help to understand or clarify this phenomenon.

This result related to Bandura’s self-efficacy theory was significant in similarity and difference. Bandura believes achieving self-efficacy is one of the most significant contributors to competence and capability. His model recommended four sources of self-efficacy, including previous activities, vicarious practices such as having a role model, verbal encouragement such as coaching and evaluative guidance, and emotional arousal [63, 64]. It refers to a nurse’s belief in his/her ability to effectively cope with tasks, duties, and encounters related to his/her proficient role [65–68]. Kim and Sim examined the structural relationship among clinical nurses’ self-efficacy, communication ability, self-leadership, and nursing performance. The results revealed that the relationship between the nurses’ communication ability and self-leadership had a statistically significant effect. Likewise, the relationship between communication ability and self-efficacy had a statistically significant effect. Third, the nurses’ communication ability affected nursing performance through self-efficacy [69].

### Application of research findings

The present study findings can be used to re-evaluate nursing education programs and CME courses in content, training methods, and evaluation of learners. Our findings will also help health policymakers improve nursing education quality and promote effective CME programs.

### Study strength and limitation

This research is a qualitative study based on the experiences and perceptions of nurses, reporting more comprehensively the experience of nurses in continuing medical education programs in promoting competency toward noncommunicable diseases in the Iranian context. Exploring the experience and perception of the nurses involved in the phenomenon and looking through their eyes can bring something new and help us comprehend the phenomenon under study. Nevertheless, this kind of qualitative research has some limitations. The main limitation of this small-scale study was the sole participation of actively employed nurses at teaching hospitals at Shiraz University of Medical Sciences. Therefore, our findings cannot be generalized to other populations or educational settings in Iran. In addition, the study did not include distance learning courses and only focused on in-person CME sessions. Therefore, the context and the setting are defined in detail, aiming to improve the judgment of transferability. Correspondingly, relating the results to theoretical concepts is an effort to enhance the transferability of the study outcomes. It is recommended that further studies should be conducted in other teaching hospitals across the country to include the experience of other nurses in the development of more effective CME courses. Semi-structured interviews used to gather the data made the results more comprehensive and subjective.

## Conclusion

Nurses spend most of their time caring for patients; thus, they play an essential role in the healthcare system. Given the high prevalence of death due to noncommunicable diseases, the nurses’ experience on the effect of continuing education is valuable.

When used as a training course, the CME program can be a valuable part of CME but cannot succeed without a needs assessment. Based on the nurses’ experience caring for patients with noncommunicable diseases, such nurses’ professional competence and performance can be improved through intrinsic motivation stimulation, planning and implementation of training programs based on professional needs, and effective assessment of the teaching/learning process.

The learning objectives and educational activities should be connected to a learning theory and clarified for the nurses. Nurses should also receive support during the course to benefit from the CME program. Continuous pedagogical interaction with feedback and reflection between nurses, managers, and instructors should be provided to enhance deep learning. CME activities are seldom evaluated in the context of their long-term impression on practice or professional improvement. It is appropriate to explore developing locally and contextually relevant models to assess the impact of these training programs. It is suggested that further studies should be conducted in the context of other disciplines and health care providers to better find out the barrier of CME program in other areas.

## Electronic supplementary material

Below is the link to the electronic supplementary material.

Supplementary Material 1

## Data Availability

The datasets produced and analyzed during the present study are not publicly accessible due to participant confidentiality but are obtainable from the corresponding author on reasonable request.

## List of abbreviations

CME: continuing medical education
NCD: Non-communicable disease
